# Experiences of participants in ETEC controlled human infection model studies

**DOI:** 10.1371/journal.pone.0339179

**Published:** 2025-12-22

**Authors:** Bettina Wunderlich, Kathryn Chang, Jessica L. Schue, Lawrence H. Moulton, Nancy Kass, Ruth A. Karron, Kawsar R. Talaat

**Affiliations:** 1 Center for Immunization Research, Johns Hopkins Bloomberg School of Public Health, Baltimore, Maryland, United States of America; 2 Department of International Health, Johns Hopkins Bloomberg School of Public Health, Baltimore, Maryland, United States of America; 3 Berman Institute of Bioethics, Johns Hopkins University, Baltimore, Maryland, United States of America; 4 Department of Health Policy and Management, Johns Hopkins Bloomberg School of Public Health, Baltimore, Maryland, United States of America; IAVI, UNITED STATES OF AMERICA

## Abstract

**Background:**

Controlled human infection model (CHIM) studies raise ethical issues due to intentional exposure of healthy volunteers to a pathogen. In this study we examine the experiences of participants who have chosen to participate in enterotoxigenic *Escherichia coli* (ETEC) CHIM studies in a single research center.

**Methods:**

An interviewer-administered paper-based survey was conducted with participants in an ETEC CHIM study at the Center for Immunization Research (CIR) from 2009–2020. We analyzed participant characteristics, study experiences, and perceived risks before and after study participation.

**Results:**

48 participants in ETEC studies were interviewed. 81.3% of participants identified as Black or African American, 43.8% completed at most high school, and 39.6% of interviewees had incomes of less than $18,500 in the last year. Most respondents enjoyed participating and were glad they joined. Participants most frequently identified interactions with research staff, compensation, and food quality as the most positive aspects of their experiences. There was a significant reduction in the perceived risk of the ETEC study after completing participation compared to perceived risk before joining.

**Conclusions:**

Despite the expected side effects of ETEC CHIM studies, most participants believe the benefits outweigh the risks and would participate again.

## Introduction

Controlled human infection model (CHIM) studies, also commonly referred to as human challenge studies, have gained significant traction over the last few years. These studies involve the planned and controlled inoculation of an infectious organism into willing and healthy adult participants [1]. CHIM studies are conducted with pathogens that cause a disease that is treatable or self-limited. Through experimental challenge of susceptible individuals, CHIMs can improve our understanding of the pathophysiology of infectious agents in humans and can be used in preliminary assessments of the efficacy of vaccines or treatment strategies [1–4]. CHIM studies can produce key safety and efficacy data quickly, and can be used in vaccine development for proof-of-concept, vaccine prioritization, immunogenicity, or disease pathogenesis studies [3–6].

There are potential risks from deliberate infection to be considered. Symptoms and expected side effects depend on the causative agent; in some CHIM studies participants have minimal symptoms (as is the case with dengue or Zika challenge studies) and may only require evaluation in outpatient settings [7]. On the other hand, particularly with enteric challenge studies (such as enterotoxigenic *Escherichia coli* (ETEC), *Shigella*, etc.), participants might experience significant symptoms, including diarrhea, vomiting, and fever, and most participants must stay at an inpatient facility to monitor symptoms and prevent transmission of the infectious agent to the community [8,9]. In addition to the potential side effects caused by the pathogen, there is also risk associated with the experimental intervention (vaccine, therapeutic, or preventative medication) and treatment (i.e., antibiotic) in a CHIM study.

Due to this intentional exposure of healthy volunteers to a pathogen and its associated symptoms, as well as potential risks associated with experimental interventions and treatments, with no prospect of direct benefit to the participants, CHIM studies raise considerable ethical issues [5,10,11]. In particular, questions are raised on whether the individual risks outweigh indirect and societal benefits in these studies, and if consent is sufficiently voluntary and information properly understood to be confident about participants’ intentions, especially in a context where financial remuneration is provided. Consensus has been reached that if these studies are pursued with careful considerations about safety and informed consent, have high benefits to the community, and mitigated risks, they should continue to be conducted [5,12]. However, despite the ethics of these trials being discussed among investigators and ethics committees [1], there is limited literature on how participants perceive healthy volunteer studies [13–16], and less so for CHIM studies [17]. This study was designed to begin to fill this gap by examining the experiences of participants who elect to enroll in enterotoxigenic *Escherichia coli* (ETEC) challenge studies.

## Materials and methods

### Study design, sampling and data collection

A structured interviewer-administered paper-based survey was conducted with prior participants in CHIM and other inpatient studies at the Johns Hopkins Center for Immunization Research (CIR) between 18/Dec/2009 and 05/May/2020. To be eligible, participants’ last study had to have been completed more than 3 months but no more than 15 months before the structured interview. Participants could only be interviewed once, irrespective of how many studies they had enrolled in. Given the small study sizes, a population census of eligible participants was used to recruit volunteers. This survey was in-person or telephone-based, depending on the interviewee’s preference. Interviews took approximately 45 minutes to complete. Interviewers were trained prior to conducting the survey to increase consistency of interviews and were not involved in the inpatient studies in order to preserve distance between the participants and study team and decrease the potential impact of social desirability bias. The data included in this analysis are from those volunteers who self-reported having last participated in an ETEC CHIM study.

### Ethics

This study was approved by the Johns Hopkins Bloomberg School of Public Health Institutional Review Board (IRB # IRB00002353). Oral informed consent was obtained from all participants involved in the study, documented by signature, date, and time of person obtaining consent on an oral consent script.

### Statistical analysis

Descriptive statistics regarding participant characteristics, their study experiences, and the perceived risks before and after their ETEC study at the CIR were analyzed. A nonparametric Wilcoxon signed-rank test assessed change in risk perception. StataIC version 16 and R version 4.2.1 were used to conduct all data analysis.

## Results

### Participants

Out of 152 CIR participants who were interviewed, 48 (31.6%) were participants who had last participated in an ETEC inpatient study. As illustrated in Table 1, 56.2% of these participants were male, 37.5% were 25–34 years old, 81.3% of participants identified as Black or African American, 43.8% completed at most high school, and 39.6% of interviewees earned less than $18,500 in the preceding year. 60.4% of participants were employed at the time of the interview and the same percentage had medical insurance. When asked about prior clinical trials (inpatient or outpatient), just over 20% of participants said this was the first study they participated in, 60.4% of participants had participated in 2–5 studies, 10.4% in 6–10 studies, and 8.3% had participated in over 10 studies.

**Table 1:** Characteristics of participants in ETEC challenge studies at the CIR between 2010-2020.

Characteristic	Participants (N = 48) *no. (%)*
*Sex*	
Male	27 (56.2)
Female	21 (43.8)
*Age at interview*	
18-24	7 (14.6)
25-34	18 (37.5)
35-44	17 (35.4)
45-55	6 (12.5)
*Race*	
Black or African American	39 (81.3)
Mixed Race	1 (2.1)
White	5 (10.4)
Other	1 (2.1)
Refused	2 (4.2)
*Education*	
Less than high school	1 (2.1)
Some high school	3 (6.3)
High school graduate/GED	17 (35.4)
Some college	16 (33.3)
College graduate	5 (10.4)
Some graduate school	4 (8.3)
Postgraduate/professional degree	2 (4.2)
*Income*	
Under $18,500	19 (39.6)
$18,500-$34,738	12 (25.0)
$34,738-$55,331	8 (16.7)
Over $55,331	1 (2.1)
Don’t Know	4 (8.3)
Refused	4 (8.3)
*Employed*	
Yes	29 (60.4)
No	19 (39.6)
*Medical Insurance*	
Yes	29 (60.4)
No	19 (39.6)
*Total number of studies participated in*	
1 (this one)	10 (20.8)
2-5	29 (60.4)
6-10	5 (10.4)
>10	4 (8.3)

### Best and worst aspects of participating

**Fig 1:**
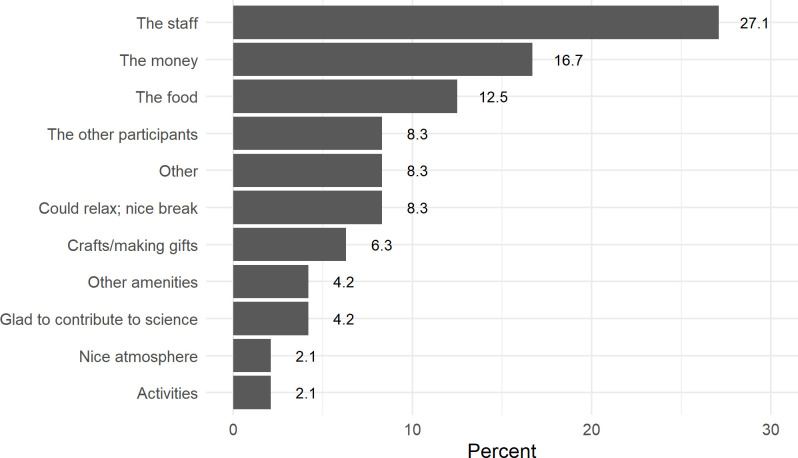
Best aspect of participating in CIR ETEC inpatient study.

When asked what the best thing was about participating in their most recent ETEC trial at the CIR, 27.1% of participants said the staff were the best part (Fig 1), followed by money at 16.7%, and food at 12.5%.

**Fig 2:**
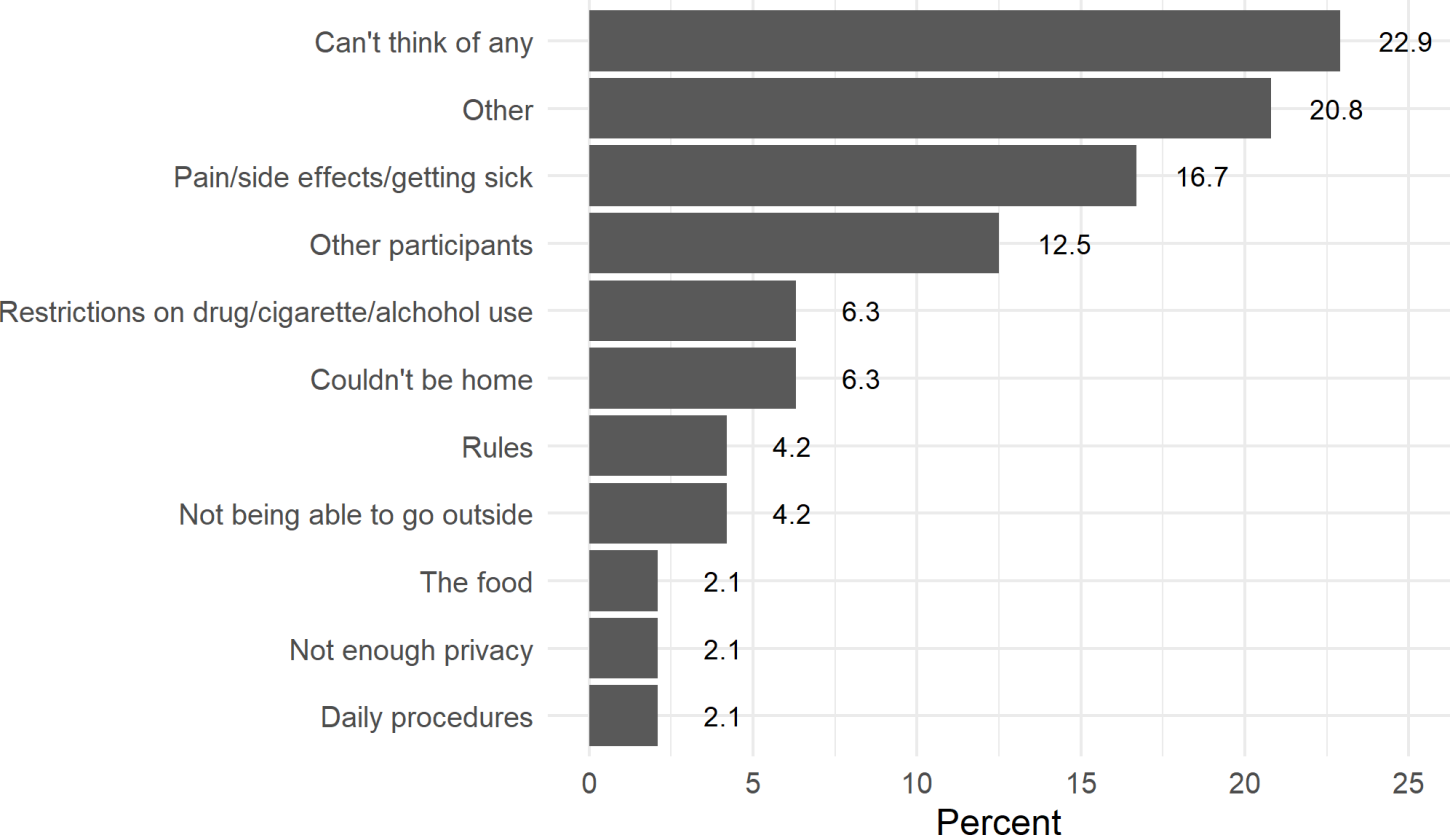
Worst aspect of participating in CIR ETEC inpatient study.

When asked about the worst aspect of participation (Fig 2), 22.9% of participants said they couldn’t think of any bad aspects to participating. This was followed by 16.7% who said the worst part was the side effects, and 12.5% said it was the other participants. 20.8% of participants said “other”, which included not enough bathrooms, wanting to receive compensation as a lump sum, having to fast, potentially getting addicted to making “easy money”, noise, and the presence of a security guard.

### Other participant perceptions

**Fig 3:**
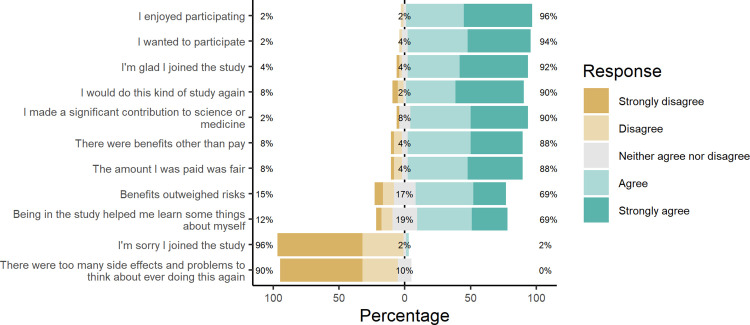
Responses to Likert questions on perceptions of participating in ETEC studies.

As shown in Fig 3, most participants either agreed or strongly agreed that they enjoyed participating (96%), that they wanted to participate in their most recent study (94%), that they were glad they joined the study (92%), and that they would do this kind of study again (90%). In terms of the benefits, 90% agreed or strongly agreed that they made a significant contribution to science or medicine, that there were benefits other than pay (88%), that the pay they received was fair (88%), that the benefits outweighed the risks (69%), and that they learned some things about themselves being in the study (69%). 2% of participants agreed or strongly agreed that they were sorry they joined the study, and no one who completed the survey stated that there were too many side effects and problems to think about ever doing this again.

### Risks perceived before and after participating in studies

**Fig 4:**
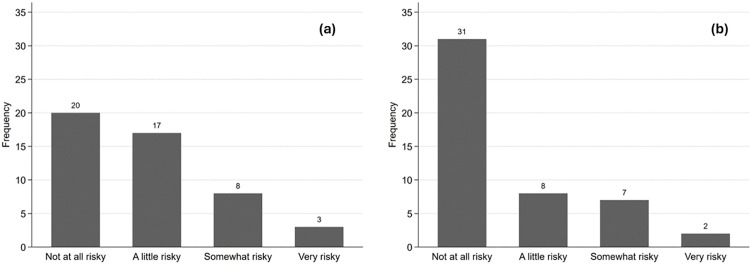
(a) Recalled risk level perceived by participants before ETEC study at the CIR; (b) Risk level perceived by participants after ETEC study at the CIR.

**Fig 5:**
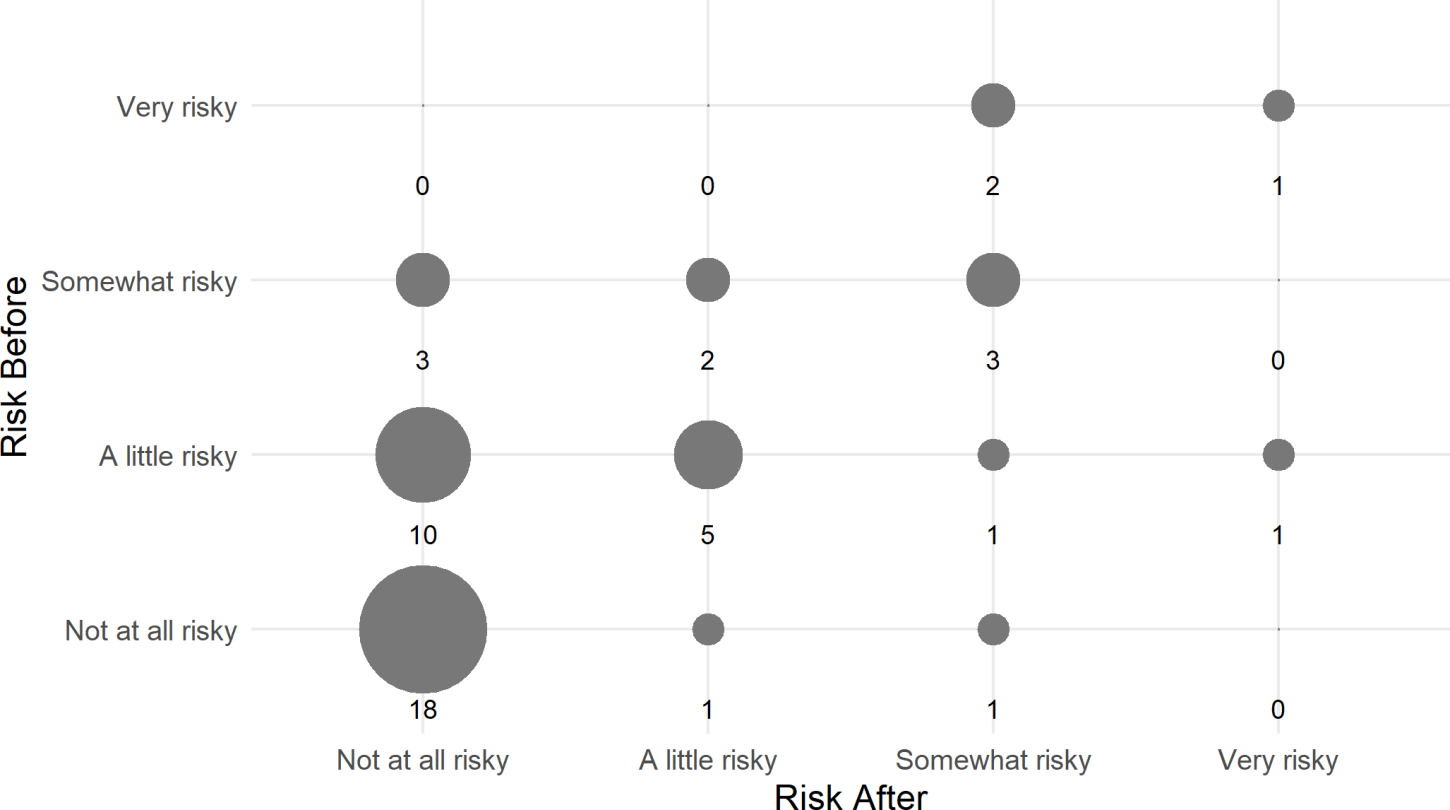
Change in recalled perceived risk before and after participation in ETEC CHIM study.

Participants were asked retrospectively about how risky they had thought the ETEC CHIM study was before and after participation, on a scale from “Not at all risky”, “A little risky”, “Somewhat risky”, to “Very risky”. When asked how risky they thought the study would be before they participated, 20 (41.7%) thought it was “Not at all risky”, 17 (35.4%) considered it “A little risky”, 8 (16.7%) said “Somewhat risky”, and 3 (6.2%) “Very risky” (Fig 4 (a)). Following participation, 31 (64.6%) thought it was “Not at all risky”, 8 (16.7%) considered it “A little risky”, 7 (14.6%) said “Somewhat risky”, and 2 (4.2%) “Very risky” (Fig 4 (b)). How participants perceived risk before and after is shown in Fig 5. Of the participants who found it “Somewhat risky” or “Very risky” prior to the study, 4 were first-time participants and 7 were participants who had been in 2–5 studies (S1 Table). None of the participants with more experience (≥ 6 studies) thought it was “Somewhat risky” or “Very risky” prior to the study, although one person thought it was “Somewhat risky” after participation.

Risk perception did change after completing the study. Of the 48 respondents, 17 (35.4%) moved towards feeling the study was less risky, and 4 (8.3%) moved towards more risky. The null hypothesis of no difference between before and after was rejected (p = .007, Wilcoxon matched-pair signed rank test).

### Advice to a friend

Participants were also asked what advice they would give a friend who saw an ad for one of the CIR inpatient studies and was thinking of joining for the first time. Responses varied; some said they would urge their friend to join and go for it. Others also brought in the perspective of doing good by participating. Some participants were more practical and discussed logistical considerations like what to pack, impact of confinement, and lack of privacy. Still others took a more cautious approach mentioning that these studies are not for everyone and for those interested to thoroughly know what they are signing up for. Select relevant quotes are displayed in Table 2.

**Table 2:** Participant’s advice they would give to a friend about a CIR inpatient study.

Advice	Quotation
*Go for it*	“I would highly recommend they do a study there.” *– Participant 31*
	“I would tell them to do it, it’s a great experience.” – *Participant 39*
*Doing good*	“I would tell them it would be a great idea and they would be doing something good for someone.” *– Participant 12*
	“It can better the world, another country, but also benefits you money-wise.” *– Participant 13*
	“I have tried to point people in this direction if they needed extra money and wanted to do something positive.” *– Participant 34*
*Logistics*	“Pack light.” *– Participant 4*
	“I would just want them to be prepared to be confined for whatever period of time if they weren’t used to it, or if they are the type of person who wanted to be able to go outside. I really didn’t miss being able to do that but some people would be uncomfortable with it.” *– Participant 30*
	“I would let them know they would have to share a room with 4 or 5 people and that they are real strict on a lot of the things that you bring in.” *– Participant 33*
*Know what you are signing up for*	“I would tell them to read up on everything, what are the symptoms, what is the study for before you enter.” *– Participant 9*
	“I would tell them to at least go to the screening and ask a lot of questions to get all the information they can and for them to decide for themselves if it is something they want to do. It’s not for everybody.” *– Participant 16*
	“I would say ask a lot of questions, ask the doctors questions. Maybe Google yourself and look around what it is they are putting into you and why.” *– Participant 19*
	“Ask questions, find out what exactly is it for, if it’s ever been tested before. That way you at least know the side effects. Don’t go in blind.” *– Participant 26*
	“Do the homework before you participate. Make sure you are comfortable with whatever is being said. Read the consent, ask questions, don’t look at money as the sole price; at the end of the day, if you are uncomfortable with it, money is nothing.” *– Participant 27*

## Discussion

This study provides the perspective of participants in ETEC CHIM studies at the Center for Immunization Research. Among participants, almost 40% earned less than $18,500 a year, were unemployed, and did not have medical insurance; the majority did not have a college degree, and more than 80% identified as Black or African American. The proportion of Black or African American adults in this study is slightly higher than Baltimore City’s distribution, where 58.4% identify as Black or African American [18]. However, the preponderance of lower income participants adds to and corroborates current research which indicates that participants in early-phase healthy volunteer studies tend to be of lower socioeconomic status when the direct benefit of the study is compensation [16,19]. In situations where there might be an alternative direct benefit (such as access to a vaccine) or when altruistic considerations are high, such as during the COVID-19 pandemic, the participant demographics in a trial has been seen to shift [20,21]. Future CHIM studies might see different participant demographics as more people have the ability to work remotely following COVID-19, and as a result of the promotion of clinical trials by organizations such as 1Day Sooner [22].

This survey study found that aside from money, the food and the staff were considered the best aspects of participating, the latter ranking higher than compensation. As in previous work, when asked about the worst aspect of participating, almost one quarter reported they couldn’t think of any (22.9%) [15]. 16.7% reported the side effects experienced and 12.5% reported the other participants as the worst aspect of the study. Thus, participant interactions with other people including staff and study participants — especially since CHIM studies last several days to weeks — are important to consider when enrolling a cohort, as this plays a strong role in the overall experience of the participant. Our findings validate those of other studies, which have also found that interactions with other people are important in shaping participants’ experiences in similar healthy volunteer studies [15] reinforcing the importance of allocating extra time and effort to ensuring a good environment for the study, especially one which requires long periods of confinement.

We found that in general, participants enjoyed participating in the studies (96%) and perhaps more importantly, they wanted to participate (94%). 92% said they were glad they joined the study and 90% said they would do this kind of study again. Additionally, 90% thought that they made a significant contribution to science or medicine, 88% agreed that there were benefits other than pay, and 69% reported that they learned things about themselves through participating. Thus, despite the fact that many people develop symptoms during the ETEC CHIM, there seems to be a general positive view of participating. Volunteers wanted to participate, would do so again, and found purpose in participating. In fact, for almost 80% of participants, this was not their first research study, and 8% had participated in over 10 studies. 88% of participants also thought that the pay they received was fair, which is an important consideration when trying to avoid undue influence from payments that are too high but also avoid exploitation of participants through low payment [23]. The latter is especially true with CHIM studies because they can be particularly burdensome to healthy adult participants due to intentional infection and the long confinement required [24].

Participants in CHIM studies face many potential risks: those associated with experimental vaccines, risks of exposure to a pathogen, risks of the study procedures, risks associated with the challenge treatment as well as treatment failure, and potential psychological and social harm [25]. A literature review of 308 CHIM studies spanning four decades (1980–2021) conducted by Adams-Phipps et al. found that of 15,046 participants, there were 24 reported serious adverse events, but no reported deaths or cases of permanent sequelae [26]. Despite the real and inherent risk in a study where a significant proportion of volunteers become ill, perceptions of the risks of participating in these studies are also significantly diminished after study completion. When asked after the studies were over their recollection of how risky it seemed to them before the study started, 20 (41.7%) participants considered it not at all risky, and this jumped to 31 (64.6%) following their participation. It is important, however, to consider the inherently subjective nature of risk perception and the role of potential recall limitation due to the retrospective nature of this study and positive study outcome [27]. Risk perception is a person’s subjective assessment of how likely an unfavorable outcome will take place [28]. As stated by Slovic et al., one of the most important underlying assumptions in their psychometric paradigm framework is that risk is inherently subjective and people have invented this concept to better understand and manage the dangers and uncertainties of life [29]. Nevertheless, risk perception is still commonly found in scientific literature and closely tied to integral affects or emotions during decision-making [30,31]. As in this study the perceived risk before and after participating were assessed at the same time following participation completion, their assessment of risk prior to the study may not be accurate. However, prior research has shown that emotions can be recalled fairly accurately, and that their recall may be distorted by their current emotional state (which in this case is weighted towards less risky), suggesting that the difference in risk perception may be even greater [32]. This significant difference in risk perception could imply that there is much unknown risk before the study and that the environment is controlled to minimize potential risks during the inpatient stay [24]. Jao et al. found that controlled human malaria infection studies were mostly unfamiliar to the lay public in low- and middle-income countries, and similarly this might also be the case for CHIMs in higher income countries, potentially increasing the level of initial perceived risk [17]. This is strengthened by the fact that participants who considered the study to be “Somewhat risky” or “Very risky” before the study were those with less clinical trial experience (≤5 studies). In future studies, it would be interesting to look at the perceived burden these studies have on participants, not just whether participants consider themselves at risk. Furthermore, as the word “risky” might be interpreted differently among participants, such wording should be clearly defined in future surveys to increase interpretability.

When asked about what advice they would give a friend who found out about a study, many seemed to have a positive perspective on these trials and would recommend participating. Some mentioned compensation while several others described this as an opportunity that can help others as well. While nobody was strongly against participating, many would recommend ensuring that their friend understood what they were signing up for and what the study entails. As stated by one participant, it is not for everyone, and knowing the details of why it is being done, what is already known, and what can be expected is paramount for these studies. This stresses the importance of the informed consent process and finding ways to communicate the most essential information in an easily digestible format so that participants truly understand the requirements, procedures and risks of study participation.

This study was able to assess the experiences of participants in several ETEC inpatient studies at the CIR and provide valuable information that builds on the small body of existing literature. Nevertheless, there are limitations to the study. First, as only people that participated in CIR ETEC studies were analyzed, some of the results, in particular the best and worst aspects and their overall experiences, may not be generalizable to other locations or to challenge studies with other pathogens. The generalizability is further hindered by the small sample size (48 participants), and that most participants had prior clinical trial experience; this was the first study for only 10 participants. There may also have been non-response selection bias, where participants who could not be reached or decided not to complete the survey were systematically different, and perhaps had worse experiences, than those who did. Another important consideration is social desirability bias; as this was an interviewer-led survey, respondents may have tailored their answers according to social norms and answered what they thought interviewers wanted to hear instead of what they actually believed. Lastly, as this was a cross-sectional study conducted several months after study completion, asking about risk perception from the time prior to study participation was likely influenced by recall limitation and skewed in favor of current perceptions of the participant. To mitigate against recall bias, future studies could perform longitudinal surveys prior to and after study participation.

## Conclusions

Despite the side effects expected during ETEC CHIM studies, most participants were glad they chose to participate and said that they would do so again. Interactions with staff, other participants, and food quality can greatly impact participants’ perceptions of their overall experiences and should be taken into consideration when planning a study. The risk perception of participating in such a study was greatly diminished following participation.

## Supporting information

S1 Table Perceived risk before and after by number of studies. (DOCX)

S1 File Basic Study Dataset. (XLSX)
