# Supplementary material for: Experiences of participants in ETEC controlled human infection model studies
Source: PLoS One. 2025 Dec 22;20(12):e0339179. doi: 10.1371/journal.pone.0339179 (PMC12721547; doi:10.1371/journal.pone.0339179)
Supplement: S1 Table — (DOCX) [file pone.0339179.s001.docx]

|  | **Risk before (N=48)** | | | | **Risk after (N=48)** | | | |
| --- | --- | --- | --- | --- | --- | --- | --- | --- |
|  | **Not at all risky**  (*n*) | **A little risky**  (*n*) | **Somewhat risky**  (*n*) | **Very risky**  (*n*) | **Not at all risky**  (*n*) | **A little risky**  (*n*) | **Somewhat risky**  (*n*) | **Very risky**  (*n*) |
| *Number of studies* |  |  |  |  |  |  |  |  |
| 1 | 4 | 2 | 3 | 1 | 7 | 1 | 2 | 0 |
| 2-5 | 9 | 13 | 5 | 2 | 17 | 6 | 4 | 2 |
| 6-10 | 5 | 0 | 0 | 0 | 4 | 0 | 1 | 0 |
| >10 | 2 | 2 | 0 | 0 | 3 | 1 | 0 | 0 |

**S1 Table.** **Perceived risk before and after by number of studies.**
